# Congenital Hepatic Fibrosis in a 2-Year-Old Child Presenting with Fever of Unknown Origin

**DOI:** 10.1155/2023/4497784

**Published:** 2023-11-01

**Authors:** Michael P. Penfold, Wentiirim B. Annankra, Nathan C. Hull, Margarita Corredor

**Affiliations:** ^1^Mayo Clinic, Rochester, MN, USA; ^2^University of Rochester Medical Center, Rochester, NY, USA

## Abstract

Congenital hepatic fibrosis is a rare, autosomal recessive, fibro-polycystic disease resulting from ductal plate malformation, leading to proliferation and fibrosis of bile ducts. Progressive hepatic fibrosis leads to portal hypertension and varices which can present with life threatening gastrointestinal hemorrhage. We report a case of congenital hepatic fibrosis in a 2-year-old child who presented with 8 days of fever without any significant medical history or physical examination findings.

## 1. Introduction

Congenital hepatic fibrosis is a rare autosomal recessive disease caused by biliary ductal plate malformation which leads to proliferation and fibrosis of the bile ducts. It may be an isolated disease or associated with other hepatobiliary cystic disease or autosomal polycystic kidney disease. The initial presentation is commonly hematemesis or melena during childhood which is the result of cirrhosis and severe portal hypertension. However, patients may have a more indolent presentation with mild transaminitis in adulthood [1].

There are only a few hundred cases of congenital hepatic fibrosis reported in the literature, and the incidence is unknown. However, in areas with high rates of consanguinity, the estimated incidence is 1 in 10,000–20,000 [2]. Definitive diagnosis requires a liver biopsy demonstrating diffuse portal and perilobular fibrosis with intact lobular structures [3]. Here, we present a case of congenital hepatic fibrosis in a two-year-old girl who presented with fever of unknown origin.

## 2. Case Presentation

A previously healthy and fully immunized 2-year-old female presented to the emergency department (ED) with eight days of intermittent fever. She was previously seen on day three of fever in the ED of an outside hospital where she was treated with Augmentin for a suspected left acute otitis media. She re-presented to the outside hospital ED on day six of fever and was noted to be well-appearing, with an unremarkable physical exam and no localizing source of infection. Laboratory testing revealed mildly elevated transaminases: ALT 37 U/L (0.62 *µ*kat/L) and AST 52 U/L (0.87 *µ*kat/L). Her CRP and ESR were elevated to 61.4 mg/dL (614 mg/L) and 51 mm/h, respectively. A CBC was entirely normal. Blood and urine cultures were obtained and did not have bacterial growth during hospitalization. SARS-COV-2 was negative. The patient was discharged with close follow-up.

**Figure 1:**
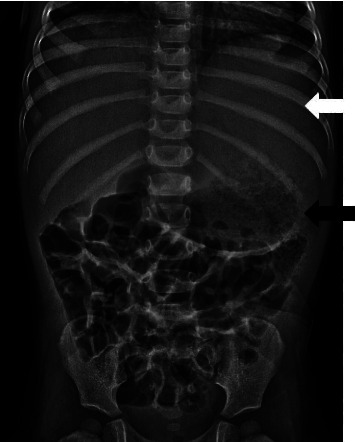
Anteroposterior supine abdominal radiograph shows abnormal soft tissue density in the left upper quadrant (white arrow) with inferior displacement of the stomach bubble (black arrow).

On day eight of fever, she presented again due to increasing irritability and decreased appetite. She had no cough, vomiting, diarrhea, rash, extremity changes, visible mucosal involvement, or conjunctivitis. Her parents denied any close contact with animals, recent travel outside of the United States, or exposures to individuals at a high risk for tuberculosis. On exam, she was sitting comfortably in her father's lap. Her physical exam was now remarkable for mild abdominal distention in the setting of an otherwise soft and nontender abdomen without palpable organomegaly. Repeat laboratory examination included a CBC which revealed an elevated white blood cell count of 18.2 × 10^9^/L (6.5–13 × 10^9^/L) with neutrophil 60% (22–51%) predominance. Urine dipstick and microscopy were negative. A metabolic panel showed resolution of previous mild transaminitis. The previously drawn blood and urine cultures continued to have no growth, and CRP remained elevated at 54.4 mg/dL (544 mg/L). An infectious work-up was initiated and was negative for Epstein-Barr virus; hepatitis A, B, C; bartonella; brucellosis; adenovirus and enterovirus. Cytomegalovirus IgG was positive and IgM negative. A blood smear was obtained, and there were no cellular abnormalities noted. An echocardiogram was performed revealing an ejection fraction of 62%, without structural abnormalities or concern for infection. An abdominal X-ray was obtained and notable for a nonobstructive bowel gas pattern with inferior displacement of the air-filled stomach, and a soft tissue density in the left upper quadrant of the abdomen. The lung bases were clear, and there were no osseous abnormalities (Figure 1).

**Figure 2:**
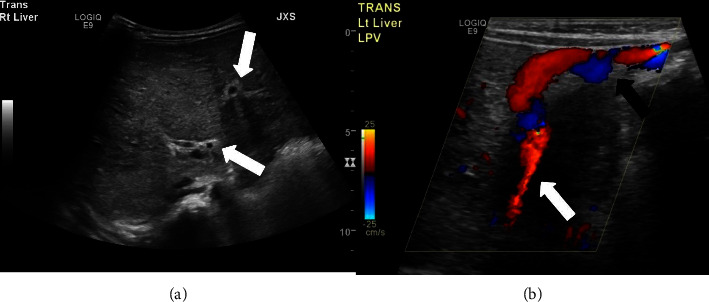
(a) Transverse grayscale image of the right hepatic lobe shows diffuse, heterogeneous, and mildly coarsened echotexture with periportal hyperechogenicity and thickening (white arrows) suggesting fibrosis. (b) Transverse ultrasound image with color Doppler shows the left portal vein (white arrow) with recanalized umbilical vein extending anteriorly to just beneath the skin (black arrow).

**Figure 3:**
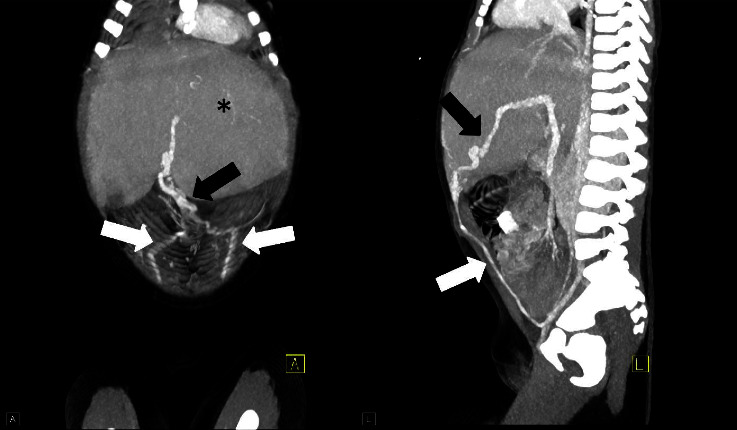
Coronal and sagittal maximum intensity projection CT with IV contrast images shows hepatomegaly with enlarged left lobe (asterisk) and recanalized umbilical vein extending from the left portal vein anteriorly near the umbilicus (black arrow) and joining the bilateral inferior epigastric veins (white arrows) to reach the iliac veins.

**Figure 4:**
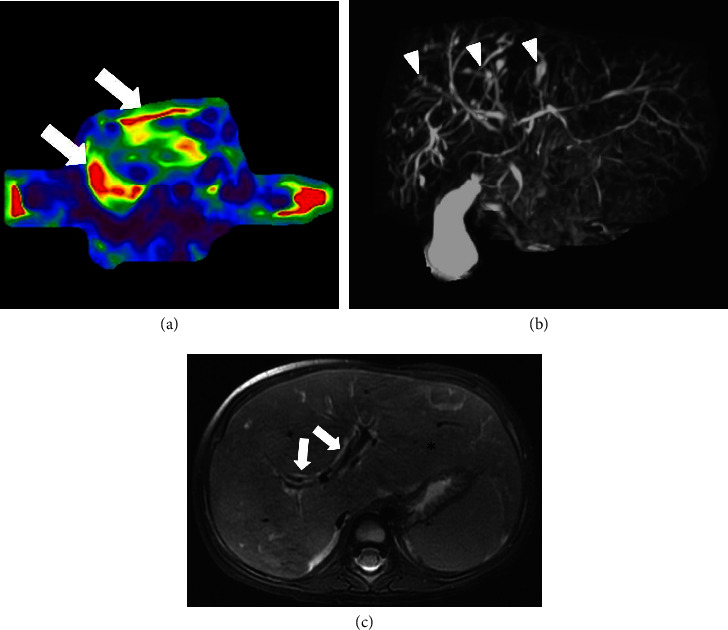
(a) Axial MR elastography image shows increased liver stiffness (white arrows) corresponding to fibrosis. (b) Coronal magnetic resonance cholangiopancreatography (MRCP) maximum intensity projection image shows scattered mixed fusiform and saccular dilatations of the intrahepatic bile ducts (arrowheads). (c) Axial T2 weighted MR image shows hepatomegaly with enlarged left hepatic lobe (asterisk) and periportal thickening and T2 hyperintensity (white arrows).

An abdominal ultrasound was then performed and revealed hepatosplenomegaly with heterogeneous echogenic liver parenchyma and a recanalized umbilical vein (Figure 2). Subsequently, abdominopelvic computed tomography with intravenous contrast was performed, which confirmed hepatosplenomegaly with enlargement of the left hepatic and caudate lobes. The scan also demonstrated signs of portal hypertension, with a diminutive right portal vein, enlarged left portal vein with recanalization of the umbilical vein, and upper abdominal varices (Figure 3). A magnetic resonance imaging (MRI) with magnetic resonance cholangiopancreatography (MRCP) and MRI elastography exam were then performed, revealing stage 3-4 hepatic fibrosis, as well as multiple ectatic intrahepatic bile ducts (Figure 4). Cumulative imaging findings suggested chronic liver disease, particularly hepatic fibrosis. An ultrasound-guided needle biopsy of the liver was performed which revealed broad bands of fibrosis and proliferation of bile ducts, characteristic findings of congenital hepatic fibrosis.

Once diagnosed with congenital hepatic fibrosis, the patient's fevers, abdominal distention, and decreased appetite were thought to be most likely explained by a cholangitis predominant presentation. Thus, she was started on Zosyn 80 mg/kg/dose IV every 6 hours for a total treatment duration of 14 days. Fevers ceased within 24 hours of antibiotic initiation, and the patient remained afebrile for the duration of her antibiotic course, with rapid resolution of inflammatory markers. After three days of antibiotic treatment, her repeat physical exam revealed a less protuberant abdomen, soft and tender to palpation, with the liver edge palpable several centimeters below the right costal margin.

**Figure 5:**
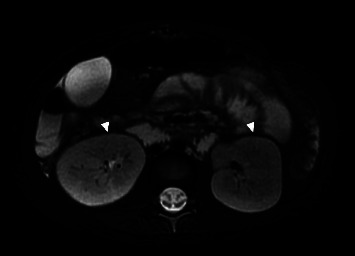
Axial T2 weighted MRI image without IV contrast shows borderline enlarged bilateral kidneys with lack of corticomedullary differentiation (arrowheads), suggesting some mild degree of autosomal recessive polycystic kidney disease.

The abdominal MRI also demonstrated borderline enlarged kidneys bilaterally, with relative lack of corticomedullary differentiation (Figure 5). Her renal function was normal throughout hospitalization.

The patient underwent genetic testing as congenital hepatic fibrosis may be associated with a wide range of underlying genetic conditions, most of which fall into the broad category of ciliopathies. A ciliopathy panel was performed which did not reveal a clear genetic explanation for her symptoms. However, she was noted to have a pathogenic variant of one *PKHD1* gene (c.4492_4494delinsAG) which is associated with autosomal recessive polycystic kidney disease (ARPKD). The patient has since returned to her baseline status, without recurrence of cholangitis after 15 months of follow-up.

## 3. Discussion

Congenital hepatic fibrosis can be isolated but is more commonly associated with other extrahepatic and hepatobiliary cystic disease including autosomal dominant polycystic kidney disease (ARPKD) and Caroli's disease [4]. Caroli's disease involves the isolated malformation of the biliary tract however, when fibrosis of the liver is present, as seen in our patient, the condition is referred to as Caroli's syndrome. Further, Caroli's syndrome may be associated with ARPKD, which can be caused by a mutation in the *PKHD1* gene. Taken together, the patient's pathogenic mutation in one *PKHD1* gene as well as of enlarged and echogenic kidneys with poor corticomedullary differentiation on imaging studies suggest a mild degree of ARPKD.

When the patients' primary clinical symptoms are related to underlying hepatic fibrosis, the presentation typically includes hematemesis or melena in childhood but can present as late as the sixth decade of life with incidental findings of transaminitis [1]. Patients presenting in the neonatal period are more likely to demonstrate a severe phenotype and are more likely to require liver-kidney transplant [1]. Because a wide range of genetic mutations can result in congenital hepatic fibrosis, there is significant variation in phenotype severity [5, 6]. Interestingly, although congenital hepatic fibrosis leads to widespread fibrosis of the liver, patients typically exhibit normal to mildly elevated AST and ALT levels with preserved hepatocellular function [1]. Despite the preserved hepatocellular function, there are characteristic morphological changes of the liver, including hypertrophy of the caudate and left lateral lobes, as seen in our patient [7]. Clinically, there may be four presentations of congenital hepatic fibrosis: portal hypertension predominant, cholangitis predominant, mixed portal hypertensive-cholangitic form, and a latent form [8]. The portal hypertension variant typically presents with painless hematochezia from esophageal and/or rectal varices, whereas the cholangitis predominant condition manifests with fever and abdominal pain. Embryologic ductal plate malformations underlie the development of congenital hepatic fibrosis, and these abnormalities are known to increase the risk of cholangitis [1]. For these patients, liver transplantation is curative, but it is reserved for the minority of patients with chronic or progressive hepatic dysfunction [9].

## Data Availability

No data were used to support the findings of this study.
